# Barriers and facilitators associated with pharmacological treatment in bipolar disorder patients

**DOI:** 10.1192/j.eurpsy.2021.519

**Published:** 2021-08-13

**Authors:** J.Á. Alcalá Partera, A. Fontalba Navas, M. Company Morales, S.L. Romero Guillena, T. Gutiérrez Higueras, F. Calera Cortés, S. Vicent Forés, L. Gutiérrez Rojas

**Affiliations:** 1 Clinical Unit Of Mental Health., Reina Sofia University Hospital., Córdoba., Spain; 2 Antequera., Northern Málaga Integrated Healthcare Area., Málaga., Spain; 3 Faculty Of Nursing, Physiotherapy And Medicine., University of Almeria., Almería, Spain; 4 Clinical Unit Of Mental Health., Virgen Macarena University Hospital., Sevilla, Spain; 5 Department Of Psychiatry., University of Granada., Granada, Spain

**Keywords:** bipolar disorder, facilitators and barriers, pharmacological treatment, beliefs

## Abstract

**Introduction:**

The main factors that are involved in a correct adherence to the therapeutic recommendations in Bipolar Disorder includes aspects related to age, sex, ethnicity, socioeconomic level and characteristics of the illness associated with the severity, comorbidity and adverse effects related to previous medicine.

**Objectives:**

To analyse the individual perception that the patient with Bipolar Disorder has regarding the positive and negative aspects of taking the recommended medication.

**Methods:**

Descriptive and interpretative observational study under the qualitative paradigm of research, extracting the data through the completion of four focus groups with ten patients everyone. To complete the codification of the content of the participant’s discourses, we rely on the QRS NVivo 10 computer program.

**Results:**

In the participant’s discourse concerning the main barriers to pharmacological treatment, for example “It’s because we live in a society and, because of that, we don’t go without medicine; if we didn’t live in society, we wouldn’t take medicine because we wouldn’t bother anyone”. Some examples of patient’s discourse, about perceived facilitators were: “I have to take medicine for my bipolar disorder, that’s it, I have a treatment, my illness has a name”.

**Conclusions:**

The main facilitators regarding the use of pharmacological treatment in Bipolar Disorder are the perceived need for treatment in the acute phase and the recognition of the illness, the shared clinical decision and the causal biological attribution in the chronic phase. About perceived barriers, social control is identified in both phases, adverse effects in the acute cases and the absence of effective treatment in the chronic state.

